# Translation and psychometric evaluation of chronic illness anticipated stigma scale (CIASS) among patients in Ethiopia

**DOI:** 10.1371/journal.pone.0262744

**Published:** 2022-01-21

**Authors:** Mohammed Hassen Salih, Lena Wettergren, Helena Lindgren, Kerstin Erlandsson, Hussen Mekonen, Lemma Derseh

**Affiliations:** 1 School of Nursing, College of Medicine and Health Sciences, University of Gondar, Gondar, Ethiopia; 2 Department of Public Health and Caring Sciences, Uppsala University and Department of Women’s and Children’s Health, Karolinska Institute, Solna, Sweden; 3 Department of Women’s and Children’s Health, Karolinska Institute, Solna, Sweden; 4 School of Education, Health and Social Studies, Dalarna University and Department of Women’s and Children’s Health, Division of Reproductive Health, Karolinska Institute, Solna, Sweden; 5 School of Nursing and Midwifery, College of Nursing and Midwifery, Addis Ababa University, Addis Ababa, Ethiopia; 6 College of Medicine and Health Sciences, Institute of Public Health, University of Gondar, Gondar, Ethiopia; Chinese Academy of Medical Sciences and Peking Union Medical College, CHINA

## Abstract

**Background:**

Stigma is common among patients with chronic illnesses. It affects the delivery of healthcare for not addressing the psychological components and may interfere with the patient’s attendance to necessary health care services. Therefore, a valid and reliable instrument to measure anticipated stigma related to chronic illness is vital to inform possible interventions. This study aimed to translate the Chronic Illness Anticipated Stigma Scale (CIASS) into the Amharic language and evaluate its psychometric properties in Ethiopia.

**Methods:**

The CIASS was translated into Amharic language using standard procedures. The Amharic version was completed by 173 patients (response rate 96%) with chronic illness from three referral hospitals in the Amhara region. Internal consistency was examined through Cronbach’s alpha. Construct validity was evaluated by confirmatory factor analysis and convergent validity by using a Pearson correlation of P-value less than or equal to 0.05.

**Results:**

The internal consistency was estimated at Cronbach alpha of 0.92. By using a structural equation model, and modification indices a model fitness testing was run and shows a root mean squared error of approximation 0.049 (90% CI, 0.012–0.075). The structural validity results in 78.8% of confirmatory factor analysis showed from the extraction of the three-dimension (components). Validity tests for convergent by using Pearson correlation positively correlated with common mental distress and negatively correlated with quality of life–BREF, and the construct validity shows a good valid tool to CIASS.

**Conclusion:**

The Amharic language version of the chronic illness anticipated stigma scale shows a satisfactory level of reliability and validity on different psychometric measures of assessment. The tool may be useful for future researchers and patients with chronic illness in the Amharic-speaking population. Moreover, it will be used to see the psychological burden related to chronic illness and for comparison among international population groups.

## Introduction

Stigma is an attribute that labels a person in discriminatory ways. Erving Goffman (1963) classically defined stigma as an “attribute that is deeply discrediting”. Stigma is the sign that indicates something out of normal about the person [1]. In 2001, Link and Phelan expanded the stigma model to include loss of social status in addition to discrimination experiences [2]. It is shifting from the ‘kingdom of the well’ to the ‘kingdom of the sick [3].

There are three types of stigma. Namely, enacted, anticipated, and perceived stigma. Anticipated stigma is the perception/belief of the individual that prejudice, discrimination, and stereotyping will likely occur in the future [4]. In other words, it is an anticipation of the enacted stigma [5]. In the case of internalized stigma, the affected individual has internalized negative stereotypes or negative attitudes, may feel ashamed or guilty because of their condition [6–10].

Anticipated stigma has been associated with undesirable health outcomes such as poor adherence to their prescribed medication, loss of attachment for their follow-up, and poor progress to their medical recovery [11, 12]. People living with chronic illnesses who had stigma experienced by healthcare workers will anticipate greater stigma from healthcare workers and in turn, it will lead to less access to healthcare [13].

Different types of stigma measurements have been developed; the stigma scale for chronic illnesses 8 item version (SSCI-8) to assess stigma in patients with neurological disorders [14], the stigma of mental illness [15], the discrimination and stigma scale (DISC-12) [16], the Internalized Stigma of Mental illness Inventory- 9 Version (ISMI-9) [17] and the CIASS-12 developed by Earnshaw and coworkers [4].

Stigma may be experienced and affect a person in different forms (physical and psychological) and we will now describe the translation process and psychometric evaluation of the Amharic language version of the chronic illness anticipated stigma scale. The translation of appropriate tools related to anticipated stigma applicable for patients with chronic illness as important steps to assess anticipated stigma among patients with chronic illness.

The CIASS was developed to measure anticipated stigma (i.e., expectations of prejudice, stereotyping, and discrimination) among people living with chronic illnesses. It is expected to measure the experience of discrimination, prejudice, and stereotyping from others in the future [4].

Measures of anticipated stigma are very important if they take specific sources of stigma into account. However, most stigma scales and tools ignore the sources of the stigma only asking about the extent to which they anticipate stigma from others in general, it is experienced rather than from specific groups; such as family members, employers, or healthcare workers. The CIASS was developed and tested psychometrically in different communities and languages, among them, the English original version, Persian version, and the Spanish Version where recent one [4, 18, 19].

At the level of our search, there is no published psychometrically validated scale to measure anticipated stigma associated with chronic illness in the Amharic-speaking population in Ethiopia. Therefore, translating and evaluating the psychometric properties of the Amharic version of the anticipated stigma scale will be important for other researchers for continuous management and follow-up among patients.

This study was aimed to translate the Chronic Illness Anticipated Stigma Scale (CIASS) into the Amharic language and evaluate its psychometric properties in Ethiopia.

## Methods and materials

### Study area, and period

The study was conducted in Amhara Region from December 2020 to 15 January 2021. The region has 5 Referral Hospitals and among those, we selected three referral hospitals for the study.

### Population, sample size, and sampling procedure

Patients who were on follow-up visits with a diagnosis of chronic illness in Amhara Regional State Referral hospitals constituted the population to be studied. Adult patients whose age was 18 or above, who were at least for one-month follow-up in chronic illness clinic, or correctly to respond to the cognitive criteria for mental illness clinic [20] were included in the study. However, acutely sick and admitted patients were excluded.

The terms *chronic illness* and *chronic disease* are often used interchangeably in clinical areas and health services programs. However, they convey different meanings that require clarification [1]. *Chronic illness* is the personal experience of living with the condition that often accompanies chronic disease [2, 3]. *Chronic disease* is defined based on the biomedical disease classification and it requires continuous management over a long period. In our paper, we included cardiovascular and respiratory disorders, cancer, diabetes, asthma, long-term viral conditions such as Hepatitis C, communicable diseases, for example, HIV/AIDS, and mental health disorders [4–6].

Based on different scholars, the sample size required for tool validation ranges from 5 to 20 patients per item [21]. The CIASS encompasses 12 items and by considering 15 patients per item, a total of 180 patients attending chronic illness clinics were approached.

Patients accepting participation were interviewed by a research assistant with a Master’s degree and BSc in health (Nursing).

The study participants were selected based on the weekly patient load in the chronic illness follow-up clinic in the three Amhara Region Referral Hospitals (University of Gondar Comprehensive Specialized Referral Hospital, Felege Hiwot Referral Hospital, Dessie Referral Hospital). Patient identification number daily list was used as a sampling frame. With proportional allocation to each hospital, 79, 61, and 40 patients, respectively, were included in the study. Proportional allocation for each unit was also used to reach the desired sample from each chronic illness follow-up clinic but not less than 5 patients in each unit.

Before the main data collection, 13 patients living with chronic illness were pretested for cognitive debriefing. For this interview patients were purposively selected considering their socio-demographic and clinical conditions.

### Variables and measures

The CIASS is a measure developed by Earnshaw and colleagues (2013). It includes 12 items divided into three subscales evaluating the extent to which the patients anticipate stigma from friends and family members, work colleagues, and healthcare workers. In family and friends sub item it contains four questions like “a friend or family member will be angry with you”. In the work colleagues’ sub-item, it included four questions like “your employer will not promote you”. And in the healthcare providers/workers sub-item, included four questions like “a health care worker will be frustrated with you”. Participant responses are indicated on a Likert-type scale ranging from 1 (very unlikely) to 5 (very likely) [4]. The average anticipated stigma score is calculated by adding up all items and averaging with the total number of items.

For the assessment of convergent validity, the Kessler psychological distress scale (K10) and the World Health Organization Quality Of Life–Bref (WHOQL-BREF) were used. Patients experiencing stigma are prone to increase psychological distress, such as depression [22]. Additionally, previous studies observed that quality of life was associated with stigma [23–25].

Psychological distress was measured by using the Kessler 10 psychological distress scales (K10) [26]. This instrument has 10 items each asking the respondent how often she/he experienced symptoms during the past 30 days and containing 5-point Likert scales (1 = never, 2 = a small part of the time, 3 = some of the time, 4 = most of the times, 5 = all of the time). The Kessler-10 scale has been validated in Ethiopia and used extensively [27, 28].

WHOQOL- BREF is a 26—item instrument consisting of four domains: physical health (7 items), psychological health (6 items), social relationships (3 items), and environmental health (8 items), and two items not included in any of the domains are an overall perception of quality of life (QOL) and general health. Each of these items was scored from 1 to 5 on a response scale, which is agreed on as a five-point Likert scale. The domain scores were calculated according to the manual for WHOBOL-BREF [29] and it was validated in the Amharic version on type 2 diabetes patients [30].

### Linguistic validation

The CIASS was translated into Amharic language using the forward-backward process of the Functional Assessment of Chronic Illness Therapy (FACIT) translation methodology [31].

The translation process included several steps: forward translation, discussion with translators, back-ward translation, expert discussion, and cognitive test on purposively selected patients.

The original English version of the instrument was translated to the Amharic language version by using forward translation to ensure semantic equivalence by two native Amharic-speaking original translators.

The two translations of the Amharic version were discussed with the main investigator (PI) to create an initial Amharic version. The initial Amharic version was then back-translated into English by two language experts (the experts are from the University of Gondar English language department).

A one-day session was held to examine the forward translated and back-translated versions by behavioral and social related experts, mental health specialists, and chronic nurse specialists with extensive knowledge of the chronic illness patient assessment for more than 16 years. They examine all of the proceeding steps and selected the most appropriate translation for each item or provided alternate translations. The overall discussion and meeting with experts were led by the primary investigator.

To determine the readability of the language used, cultural context, and to ask for their feedback on the comprehensiveness of items cognitive debriefing was conducted with a convenience sample of 13 participants at the University Of Gondar Comprehensive Specialized Hospital. During cognitive debriefing, patients were asked to say something on the overall comprehensiveness of the tool, and some modifications in some words were done. We added an explanation on the working place for those who had no experience of being employed, like in the Ethiopian context, the experiences of the farmers. In conclusion, there were no major differences from the original English language version (Table 1).

**Table 1:** The Linguistic validation process for the Amharic version of anticipated stigma scale among chronic illness patients in Amhara Region Referral Hospitals.

**Original instrument translation to Amharic**	**Forward Translator (FT1)—**He has familiarity with the literature on health-related psychology and epidemiology masters (Fluent in the Amharic, good understanding of English).
	**Forward Translator (FT2)**:—Fluent in the Amharic and Good understanding of English, but without health-related background and not informed about the tool.
**A Synthesized translated version**	**The PI and** the two forward translators assessed the two forward translated versions to identify major discrepancies.
**Back translations**	**Back translator BT1**- Language and subject expert, a good understanding of Amharic (He has familiarity with the literature on health-related, Ph. D in nursing student)
	**Back translator BT2—**Language expert in English and a good understanding of Amharic.
**Expert committee meeting for one day**	Behavioral and social related experts, mental health specialists, and Chronic nurse specialists who had extensive knowledge and experience of chronic illness patient care.
	The patient-relevant score for each item and total comprehensiveness was assessed.
**Cognitive interviews (pilot interview)**	Thirteen (13) patients were purposively selected from the chronic illness clinic at the University of Gondar Comprehensive Specialized and Referral Hospital.
**Finalized the instrument**	The principal investigator evaluated all over the content.

Key:- BT = Back Translator, FT = Forward translator, PI = Principal Investigator.

During cognitive debriefing 13 patients were interviewed by purposive sampling technique, 93.6% of the participant said it was completely relevant for the chronic illness. Their mean and SD were 2.51 & 0.5 respectively.

### Data analysis

Validity was assessed by construct and convergent validity. Construct validity was examined by Confirmatory factor analysis (CFA) by using the principal component analysis and the varimax rotation method was used. Also, scale and item analysis, and Pearson’s correlations were performed.

Convergent validity was examined by assessing correlations between the CIASS and the instruments WHOQOL BREF and the K10. Based on different scholars’ quality of life is negatively correlated to stigma and positively correlated to psychological distress.

Reliability was assessed by internal consistency. The internal consistency was calculated by using the Cronbach’s-α coefficient. Cronbach’s-α of 0.70 or greater is considered to indicate acceptable reliability [32].

Data entry at epi info V7 and exported to IBM SPSS Version 25 and STATA Version 14 was used for different psychometric test statistical analyses. All data, except identification and some socio-demographic variables, was made available at Mendeley data depository [33].

### Data quality

To maintain the quality of data collection instruments’ discussion was held with 14 data collectors and three supervisors to have a common understanding. Before the actual data collection, two-day training was given for the data collectors and supervisors.

Before the main data collection 36 patients were done for inter-rater reliability for 14 data collectors. Their result was 0.875 average measure intraclass correlation coefficient with 95% Confidence interval (0.775–0.936).

During data collection, the assigned supervisors followed each activity and gave direct support. Responses for more than 5% of the patients who had not answered, i.e. missing values of two or more questions from 12 items were excluded. But for those less than 5% correction of responses to the average score was done. The five-point Likert scale of the anticipated stigma scale was evaluated through analysis of whether all response alternatives, 1–5 were used for all items. For the total scale floor and ceiling effects were calculated [34].

### Ethical consideration

Permission was obtained from the tool developer (Dr. Valerie Earnshaw). The ethical clearance letter was received from the University of Gondar ethical review board and written consent was obtained from each study site. Because most of the participants are illiterate, the University of Gondar ethical review board approved to use of either verbal or written consent with letter reference number V/P/RCS/05/89/2020 on September 17^th^, 2020.

Before each data collection, the signature of each patient or verbal consent was secured on the first page of the consent paper. Information related to the aim of the study, voluntariness, and benefits in participating in this study was clearly stated before data collection.

The principal investigator, supervisors, and data collectors ensured the privacy and confidentiality of information throughout the study process. Information sheets and consent forms were placed in a secure place.

Confidentiality was secured at all stages and that data would be presented only for educational purposes.

## Results

### Socio-demographic characteristics of the participant

From the total 180 patients interviewed, 173 were included in the psychometric evaluation (response rate of 96%). One hundred four (60%) of the participants were male. The mean (standard deviation) age was 44 (13.8). The majority (78%) of the participants were urban residents, more than two-thirds (78%) were orthodox. Almost two-thirds (63%) of them were married. Regarding working conditions, more than two-thirds (71%) of them were working in a private or non-governmental institution. One hundred forty (80.9%) of them got information from mass media on facts related to their health, from them around two-thirds (68.9%) had got information from television (Table 2).

**Table 2:** Socio-demographic characteristics of patients diagnosed with a chronic illness in Amhara Region Referral Hospitals, Ethiopia (n = 173).

S.no	Variable	Frequency (%)
**1**	**Sex** Male	69 (39)
	Female	104 (60)
**2**	**Residency** Urban	135 (78)
	Rural	38 (22)
**3**	**Ethnicity** Amhara	165 (95)
	Oromo	3 (2)
	Others	5 (3)
**4**	**Religion** Orthodox	135 (79)
	Muslim	32 (18)
	Protestant & Catholic	6 (3)
**5**	**Marital status** Single	27 (15)
	Married	109 (63)
	Separated	13 (8)
	Divorced & Widowed	10 (14)
**6**	**Educational status** Not write & read	43 (25)
	Read and write	19 (11)
	Primary (1–8)	36 (20)
	Secondary (9–10)	32 (18)
	Preparatory and technique	21 (13)
	Diploma and above	22 (13)
**7**	**Occupation** Government	50 (29)
	Non-Gov’t & Private	123 (71)
**8**	**Information from mass-media** Yes	140 (81)
	No	33 (19)
**9**	**Source of information from Television** Yes	119 (69)
	No	54 (31)

### Clinical characteristics of the participants

More than two-thirds (72.8%) of them have fear of contagion in the hospital and the vast majority (92%) reported they attended regular follow-up.

Thirty-four patients were diagnosed with mental illness, 34 with HIV/AIDS, and 30 patients with Diabetic Mellitus. Two-thirds (67.6%) of them had a single diagnosis. The mean (SD) for the year since diagnosis was 6.9 (5.3) (Table 3).

**Table 3:** Clinical characteristics of patients with chronic illness attending follow-up clinic in Amhara Region Referral Hospitals (n = 173).

S.no	Variables	Frequency (%)
**1**	**Fear of contagion in the hospital** Yes	126 (73)
	No	47 (27)
**2**	**Regular follow up in the clinic** Yes	158 (91)
	No	15 (9)
**3**	**Comorbidity** Yes	56 (32)
	No	117 (68)
**4**	**Number of comorbidities** 1 (one)	44 (25)
	More than one	11 (6)
**5**	**Diagnosis** Hypertension	26 (15)
	CHF	9 (5)
	DM	30 (17)
	Asthma & Neurologic	5 (3)
	HIV/AIDS	34 (20)
	Cancer	20 (11)
	Mental illness	34 (20)
	Others	15 (9)

**Key:-** CHF = Congestive Heart Failure, DM = Diabetes Mellitus, HIV/AIDS = Human Immuno-Deficiency Virus/Acquired Immunodeficiency Syndrome.

### Reliability tests

#### Internal consistency

The Cronbach’s-α coefficient for the reliability of the CIASS was 0.92. Cronbach’s-α value for anticipated stigma to family and friend dimension is 0.93, to work colleagues 0.91 and to health care workers 0.88. Mean (SD) for chronic illness anticipated stigma score to family and friend was 1.88 (1.02), to work colleagues 1.86 (0.89), and for Health Care Workers (HCW) 1.6 (0.8). The floor and ceiling effect for the total anticipated stigma score was 11.6% and 0% respectively (Table 4).

**Table 4:** Internal consistency, the mean and standard deviation of dimensions of CIASS for patients with chronic illness in Amhara Region Referral Hospital (n = 173).

Dimensions of CIASS	Internal consistency (Cronbach-α)	Floor/ceiling effect (%)	Mean (SD)
**Family and Friends**	0.93	30.6/0.03	1.88 (1.02)
**Work colleagues**	0.91	0.24/0.01	1.86 (0.89)
**HCW**	0.88	0.39/0.01	1.66 (0.80)
**Total CIASS**	0.92	11.6/0	1.80 (0.75)

**Key:-** CIASS = Chronic illness anticipated stigma scale, HCW = health care workers, SD = Standard Deviation.

### Construct validity

#### Content validity

On the cognitive debriefing report, from the total of 13 participants on 12 items CIAS score, their mean (SD) was 30.1 (6). The mean (SD) score in family and friends was 11(3), working colleagues 11(3), and health care workers were 11.2 (3).

After cognitive data collection; an expert discussion was held one from psychiatry, one from chronic illness clinic nurse, and one from a clinical psychologist. Two experts were agreed on all components, whereas one expert (clinical psychologist) said “the questionnaire was nice, but I am afraid they may trigger (introduce) stigma to the people”. With the team, we discussed anticipated stigma is not a daily event and it is their anticipation. If the patient is anticipated something in their mind it needs to address before goes to psychological and physical damage. Finally, we agreed the tool can be used to assess the anticipated stigma and identify factors related to their illness.

#### Convergent validity

The final model demonstrated convergent validity with the Kessler Psychological Distress scale (K10) and WHOQOL BREF for the CIASS at 0.01 level of Pearson correlation (Table 5).

**Table 5:** Convergent validity of anticipated stigma scale among chronic illness patients in Amhara Regional Referral Hospitals (n = 173).

CIASS	Pearson correlation coefficient	
	With QOL	With K10
**Family and Friends**	-0.307**	0.577**
**Work colleagues**	-0.352**	0.449**
**HCW**	-0.271**	0.284**
**Total CIASS**	-0.375**	0.541**

** Correlation is significant at the 0.01 level (2-tailed).

**Key**:- CMD = Common mental Distress, CIASS = Chronic illness anticipated stigma scale, QOL = Quality of Life, HCW = Health Care Workers.

#### Structural validity

The KMO (Kaiser-Meyer-Olkin) sampling adequacy index on the data was 0.872 and bartlett’s test of sphericity, p-value < 0.001. After varimax rotation, the 12 items of CIASS load on three components. The first component accounted for 53.8% of the total variation, the second for 13.8%, and the third component for 11.3% of the variation. It demonstrated that the CIASS is grouped into three domains consisting of family and friends, work colleagues, and health care workers (Table 6).

**Table 6:** Exploratory factor analysis of CIASS after Varimax rotation among patients with chronic illness in Amhara Region Referral Hospital (n = 173).

Rotated Component Matrix^a^			
CIASS Questions	Component of CIASS		
	Family & Friends	Work colleagues	HCWs
Will think that your illness is your fault (Item 3)	0.868		
Will not think as highly of you (Item 4)	0.849		
Will be angry with you (Item 1)	0.819		
Will blame you for not getting better (Item 2)	0.818		
Will discriminate against you (Item 6)		0.862	
Will not promote you (Item 5)		0.859	
Will Assign a challenging project to someone else (Item 7)		0.852	
Will think that you cannot fulfill your work responsibilities (Item 8)		0.742	
Will blame you for not getting better (Item 11)			0.838
Will think that you are a bad patient. (Item 12)			0.823
Will give you poor care (Item 10)			0.809
Will be frustrated with you (Item 9)			0.774

**N.B:-** Extraction Method: Principal Component Analysis (PCA).

Rotation Method: Varimax with Kaiser Normalization.

a. Rotation converged in 5 iterations.

Key:- CIASS = chronic illness anticipated stigma scale, HCW = health care workers, PCA = principal component analysis.

**Fig 1:**
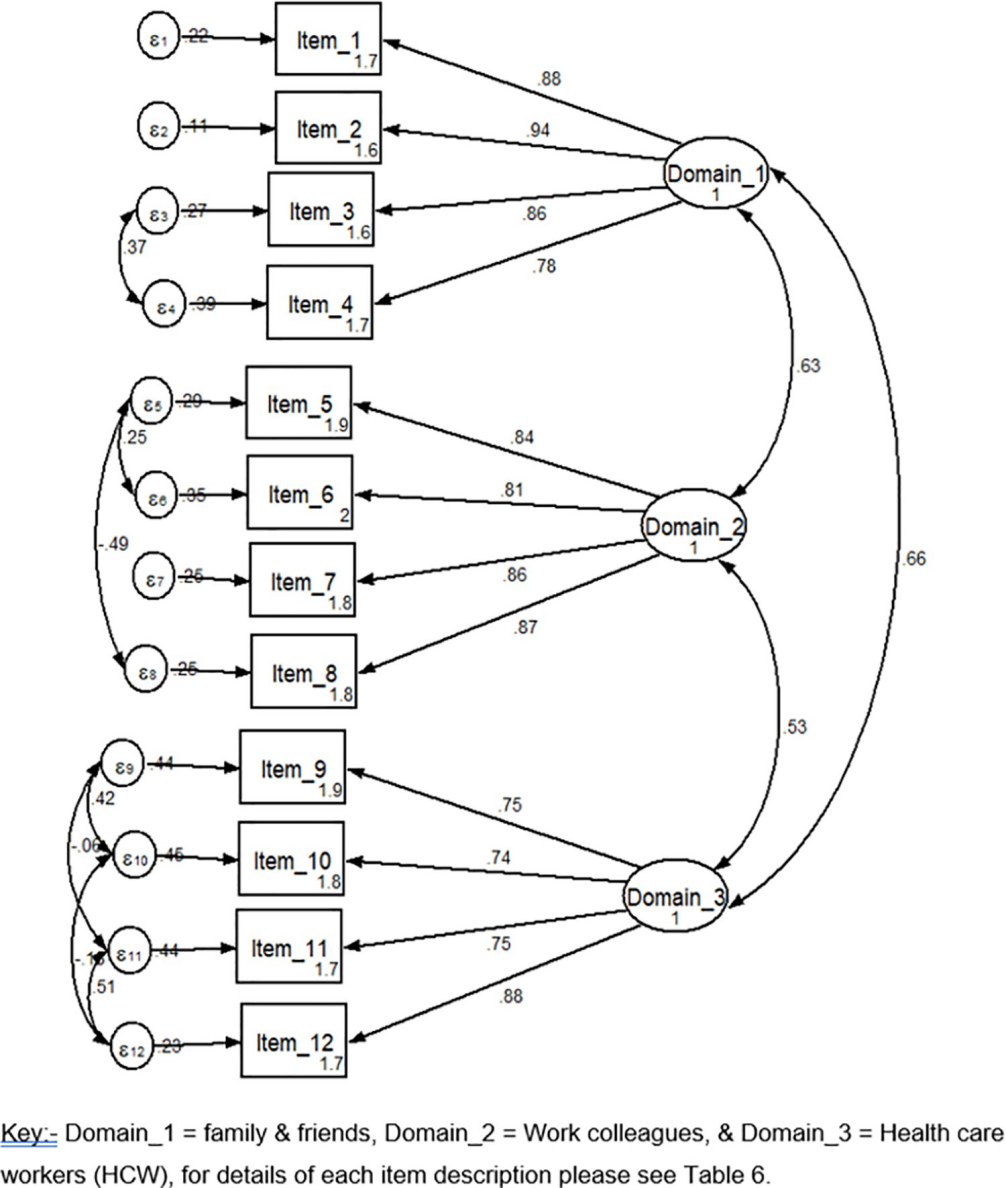


**Fig 2:**
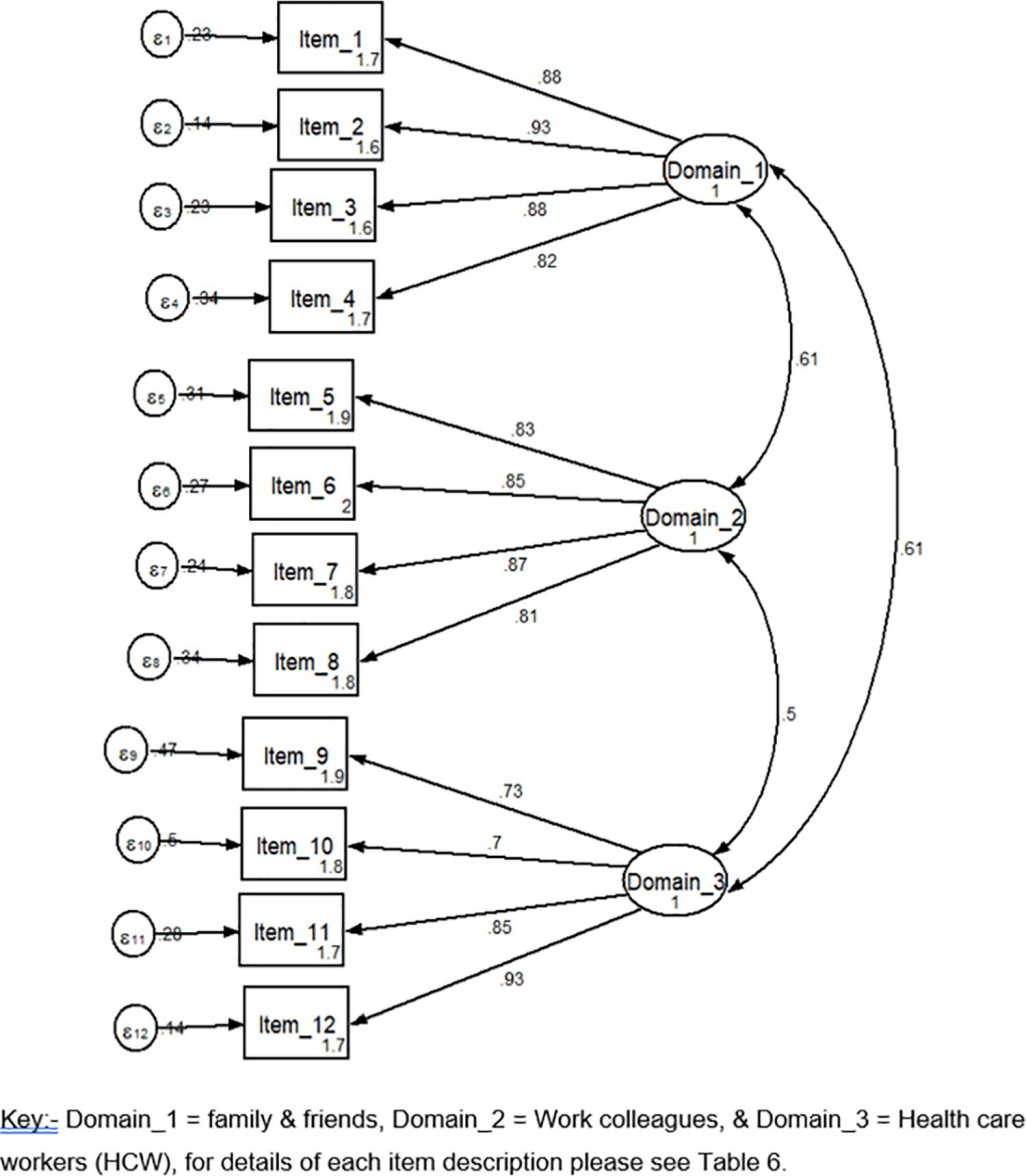


By using a structural equation model (SEM), the estimation equation yields all over model fitness with a root mean squared error of approximation (RMSEA) 0.12 (90% CI, 0.10–0.14), on baseline comparative fit index (CFI) of 0.92 and Tucker-Lewis Index (TLI) of 0.90. After this result, all over modification indices were run and gives a RMSEA 0.049 (90% CI, 0.012–0.075) CFI and TLI 0.98 and 0.99 respectively (Table 7 and Figs 1 and 2).

**Table 7:** Structural equation model estimation overall fitness results before and after modification indices for anticipated stigma scale among selected patients in Amhara Region Referral Hospitals (n = 173).

Fit statistics	Value		Description
	Before	After	
**Likelihood ratio** Chi ^2^ –ms (44)	178.418	61.954	Model vs. saturated
P > Chi ^2^	0.000	0.038	
Chi ^2^-bs (66)	1707.384	1707.384	Baseline vs saturated
P > Chi ^2^	0.000	0.000	
**Population error** RMSEA	0.120	0.049	Root mean squared error of approximation
90CI, lower bound	0.101	0.012	
upper bound	0.140	0.075	Probability RMSEA < = 0.05
P close	0.000	0.508	
**Information criteria** AIC	4504.834	4402.369	Akaike’s information criterion
BIC	4627.812	4547.421	Bayesian information criterion
**Baseline comparison** CFI	0.922	0.989	Comparative fit index
TLI	0.900	0.994	Tuckler- Lewis index
**Size of residuals** SRMR	0.054	0.034	Standardized root mean square residual
CD	0.999	0.998	Coefficient of determination

Key:- AIC = Akaike’s Information Criterion, BIC = Bayesian Information Criterion, CD = Coefficient of determination, RMSEA = Root Mean Square Error of Approximation, TLI = Tuckler-Lewis Index.

## Discussion

There are a dozen thousands of psychometric tests and tools claiming to measure an unknown character or hidden ability related to health. Anticipated stigma related to chronic illness measurement was first introduced by professor Valerie Earnshaw and her team among a variety of chronic disease patients [4]. This original English CIASS measure is a brief measure with good psychometric properties, which were confirmed in the USA, Iran’s, Colombian, and Italian’s validation studies [4, 18, 19, 35].

The study aimed to translate, culturally adapt the CIASS into Amharic language and investigate the psychometric properties of translating the version in patients with chronic illness in Ethiopia. The results support the reliability (i.e., internal consistency), and validity (i.e., structural, construct, and content) of the chronic illness anticipated stigma scale of the Amharic version.

Our finding demonstrated an excellent value of Cronbach’s α coefficient for the reliability of the scale (0.92), and it was almost in line with the study conducted in the United States of America (USA) (Cronbach’s alpha of 0.95) for the original English version tool development [4], and Iran’s study (Cronbach’s alpha of 0.88) for Persian version of anticipated stigma scale [18]. The main reason for the observed consistency could be due to the use of a standard method of language translation and following each step of translation and psychometric test daily. Our finding was higher than in the study which was conducted among Colombian Spanish-speaking patients (Cronbach’s alpha of 0.81) [19]. The main reason for the discrepancy could be due to our sample incorporating almost all chronic disease type patients.

Different research had shown that anticipated stigma affects the physical and mental well-being of people living with chronic illnesses [9, 13]. Therefore, we verified the convergent validity of CIASS relative to other constructs that are related to mental health and quality of life. The convergent validity translated tool was positively correlated with Kessler psychological distress scale. The possible reason for directly related correlation was due to those patients who scored a high level of psychological distress on the K10 tool may have a high level of anticipated stigma [22]. Also, it was negatively correlated with WHO QOL BREF, based on different types of evidence, those who score high levels of QOL may have a low level of anticipated stigma [23, 24]. These correlated findings were supported by the Spanish and Persian versions of CIASS validation studies [18, 19]. The possible reasons for similarity may be we followed the original English version tool developer suggestion and recommendation in the translation and cultural adaptation process.

Structural validity by using confirmatory factor analysis showed three components of anticipated stigma with the total score covers 78.8% data variance. The scale includes three factors; namely, anticipated stigma from family and friends, work colleagues, and health care workers. Our finding was similar to the Persian version anticipated stigma tool and the English version of the anticipated stigma tool [4, 18]. The tool was developed based on the concept related to anticipated stigma and it was more similar to the previous findings. However, our finding was higher than the study conducted among Colombian Spanish-speaking patients (a 3-factor structure explained 61.8% of the variance) [19]. The possible reason for the discrepancy may be in our study we incorporated all chronic illnesses, including HIV/AIDS and Mental illness. Therefore, we may fit a more homogenous population for our study.

This Amharic language version of chronic illness anticipated stigma shows a good model fit in RMSEA (0.049). RMSEA is one of the absolute indexes that describe closeness to fit. Values below 0.05 indicate a good model fit [36]. On other indicators of model fitness in CFI (0.98) and TLI (0.99), it was a good model fit for the study population. Based on different scholars; values above 0.95 indicate a good model fit [37–39]. Our finding was almost in line with the study conducted among Colombian and the original English version studies [4, 19]. Following the recommended method of psychometric analysis may lead to similar findings with others.

To the best of our knowledge, this is the first validation of the CIASS in the Amharic language-speaking population. Following standard procedure for the linguistic validation process, and availability of all data on Mendeley at DOI 10.17632/gvrk8njd76.1 was the strength of our study. Furthermore, the Amharic version of CIASS was evaluated among people living with a variety of chronic illnesses. Therefore, it can measure anticipated stigma among people living with a variety of chronic illnesses has an advantage.

There are several limitations of the CIASS that are important to consider. The psychometric properties of the Amharic Version CIASS were evaluated using samples of people living with chronic illnesses who reside in Ethiopia. The extent to which it is generalizable to other geographic locations is unknown. COVID-19 precaution makes us difficult to get patients repeatedly for test-retest reliability, and difficulty to get the same tool to compare convergent and divergent validity were the limitations.

## Conclusion and recommendation

This validation and psychometric study provide preliminary evidence of the Amharic version of the chronic illness anticipated stigma scale. This tool may provide specific information on the extent to which Amharic-speaking patients are anticipating chronic illness stigma and from whom. This may allow researchers and concerned organizations to better tailor stigma interventions to achieve maximum effectiveness.

Also, it may be important for an international and local researcher for comparing the magnitude of anticipated stigma among different population groups. Such type of research can trigger and easily support the researcher to see the level of anticipated stigma in the same type of population groups. Multiple language translation and psychometric tests should help communication between patients and physicians in different regions of Ethiopia, which could lead to more consistent assessment and management of chronic illness patients. Furthermore, a future researcher should continue to examine larger population groups by translating the original scale to the language of targeted population groups. Assessing the burden of stigma is about protecting and investigating together in good health and wellbeing is one of the sustainable development goals (SDG3) in Ethiopia.

## Supporting information

S1 File Questionnaire for CIASS English version. (PDF)

S1 Data Anticipated stigma tool validation main data. (XLSX)

## Acronym/abbreviation

CFI: Comparative Fit Index
CIASS: Chronic Illness Anticipated Stigma Scale
DRH: Dessie Referral Hospital
EFA: Exploratory Factor Analysis
FACIT: Functional Assessment Of Chronic Illness Therapy
FHRH: Felege Hiowt Referral Hospital
HCW: Health Care Workers
HIV/AIDS: Human Immunodeficiency Viruse /Acquired Immunodeficiency Syndrome
K10: Kessler psychological distress scale
RMSEA: Root Mean Squared Error Of Approximation
SD: Standard Deviation
SEM: Structural Equation Model
TLI: Tucker-Lewis Index
UGCRH: University Of Gondar Comprehensive And Referral Hospital
WHOQOL-BREF: World Health Organization Quality Of Life–Bref

## References

[1] Goffman E. Stigma Notes on the Management of Spoiled Identity Simon and Schuster. N Y. 1963;3.

[2] Link BG, Phelan JC. Conceptualizing Stigma. Annu Rev Sociol. 2001;27(1):363–85.

[3] Sontag S. Illness as metaphor: AIDS and its metaphors London. 1st Picador USA ed. Picador USA; 2001.

[4] Earnshaw VA, Quinn DM, Kalichman SC, Park CL. Development and psychometric evaluation of the Chronic Illness Anticipated Stigma Scale. J Behav Med. 2012/04/25 ed. 2013 Jun;36(3):270–82. doi: 10.1007/s10865-012-9422-4 22526525 PMC4370181

[5] Quinn DM, Williams MK, Quintana F, Gaskins JL, Overstreet NM, Pishori A, et al. Examining Effects of Anticipated Stigma, Centrality, Salience, Internalization, and Outness on Psychological Distress for People with Concealable Stigmatized Identities. PLoS ONE. 2014 May 9;9(5):e96977. doi: 10.1371/journal.pone.0096977 24817189 PMC4016201

[6] Lubkin IM, Larsen PD. Chronic Illness: Impact and Intervention. Jones & Bartlett Publishers; 2013. 738 p.

[7] Weiss MG. Stigma and the social burden of neglected tropical diseases. PLoS Negl Trop Dis. 2008;2(5):e237. doi: 10.1371/journal.pntd.0000237 18478049 PMC2359851

[8] Brohan E, Slade M, Clement S, Thornicroft G. Experiences of mental illness stigma, prejudice and discrimination: a review of measures. BMC Health Serv Res. 2010;10(1):1–11. doi: 10.1186/1472-6963-10-80 20338040 PMC2851715

[9] Earnshaw VA, Chaudoir SR. From conceptualizing to measuring HIV stigma: a review of HIV stigma mechanism measures. AIDS Behav. 2009;13(6):1160–77. doi: 10.1007/s10461-009-9593-3 19636699 PMC4511707

[10] Quinn DM, Chaudoir SR. Living with a concealable stigmatized identity: the impact of anticipated stigma, centrality, salience, and cultural stigma on psychological distress and health. J Pers Soc Psychol. 2009;97(4):634. doi: 10.1037/a0015815 19785483 PMC4511710

[11] Chambers SK, Baade P, Youl P, Aitken J, Occhipinti S, Vinod S, et al. Psychological distress and quality of life in lung cancer: the role of health-related stigma, illness appraisals, and social constraints. Psychooncology. 2015;24(11):1569–77. doi: 10.1002/pon.3829 25920906 PMC5029590

[12] Phelan SM, Griffin JM, Jackson GL, Zafar SY, Hellerstedt W, Stahre M, et al. Stigma, perceived blame, self-blame, and depressive symptoms in men with colorectal cancer. Psychooncology. 2013;22(1):65–73. doi: 10.1002/pon.2048 21954081 PMC6000725

[13] Earnshaw VA, Quinn DM. The impact of stigma in healthcare on people living with chronic illnesses. J Health Psychol. 2012;17(2):157–68. doi: 10.1177/1359105311414952 21799078 PMC8919040

[14] Molina Y W. Choi S, Cella D, D R. The Stigma Scale for Chronic Illnesses 8-item version (SSCI-8): Development, validation, and use across neurological conditions. Int J Behav Med. 2013;20(3):450–60. doi: 10.1007/s12529-012-9243-4 22639392 PMC3758464

[15] King M, Dinos S, Shaw J, Watson R, Stevens S, Passetti F, et al. The Stigma Scale: development of a standardized measure of the stigma of mental illness. Br J Psychiatry. 2007;190(3):248–54. 17329746 10.1192/bjp.bp.106.024638

[16] Brohan E, Clement S, Rose D, Sartorius N, Slade M, Thornicroft G. Development and psychometric evaluation of the Discrimination and Stigma Scale (DISC). Psychiatry Res. 2013 Jun 30;208(1):33–40. doi: 10.1016/j.psychres.2013.03.007 23582210

[17] Hammer JH, Tolan MD. Internal Structure and Reliability of the Internalized Stigma of Mental Illness Scale (ISMI-29) and Brief Versions (ISMI-10, ISMI-9) among Americans with Depression. Stigma Health. 2014;

[18] Nejatisafa A-A, Mozafari S, Noorbala A-A, Asgarian FS, Earnshaw VA, Sahraian M-A, et al. Psychometric Evaluation of the Persian Version of the Chronic Illness Anticipated Stigma Scale (CIASS). Int J Behav Med. 2017;24(4):634–40. doi: 10.1007/s12529-017-9645-4 28265810

[19] Trejos-Herrera AM, Vinaccia S, Bahamón MJ, Alarcón-Vásquez Y, Giraldo MR, García GG, et al. Translation and validation of the Spanish version of the Chronic Illness Anticipated Stigma Scale (CIASS) in Colombian patients diagnosed with chronic illnesses. Adv Ment Health. 2021 May 4;19(2):202–17.

[20] James BO, Omoaregba JO. Prevalence and predictors of poor medication adherence among out-patients at a psychiatric hospital in Benin City, Nigeria. Int J Psychiatry Clin Pract. 2011;15(1):27–34. doi: 10.3109/13651501.2010.519034 22122686

[21] Sousa VD, Rojjanasrirat W. Translation, adaptation, and validation of instruments or scales for use in cross‐cultural health care research: a clear and user‐friendly guideline. J Eval Clin Pract. 2011;17(2):268–74. doi: 10.1111/j.1365-2753.2010.01434.x 20874835

[22] Cantwell J, Muldoon O, Gallagher S. The influence of self-esteem and social support on the relationship between stigma and depressive symptomology in parents caring for children with intellectual disabilities. J Intellect Disabil Res. 2015;59(10):948–57. doi: 10.1111/jir.12205 26031805

[23] Alonso Caballero J, Buron Pust A, Consortium WMH. Association of perceived stigma and mood and anxiety disorders: results from the World Mental Health Surveys. Acta Psychiatr Scand 2008 118 4 305–14. 2008; 10.1111/j.1600-0447.2008.01241.x PMC3040096 18754833

[24] Vaughn-Sandler V, Sherman C, Aronsohn A, Volk ML. Consequences of perceived stigma among patients with cirrhosis. Dig Dis Sci. 2014;59(3):681–6. doi: 10.1007/s10620-013-2942-0 24248421 PMC3944010

[25] Brown RL. Perceived stigma among people with chronic health conditions: the influence of age, stressor exposure, and psychosocial resources. Res Aging. 2015/02/05 ed. 2015 May;37(4):335–60. doi: 10.1177/0164027514533133 25651574

[26] Depression scale—Kessler 10 | Health Navigator NZ [Internet]. [cited 2021 Dec 21]. Available from: https://www.healthnavigator.org.nz/tools/d/depression-scale-kessler-10/

[27] Alemayehu M, Wubshet M, Mesfin N, Tamiru A, Gebayehu A. Health-related quality of life of HIV infected adults with and without Visceral Leishmaniasis in Northwest Ethiopia. Health Qual Life Outcomes. 2017;15(1):65. doi: 10.1186/s12955-017-0636-6 28851361 PMC5576231

[28] Tesfaye M, Hanlon C, Wondimagegn D, Alem A. Detecting postnatal common mental disorders in Addis Ababa, Ethiopia: Validation of the Edinburgh postnatal depression scale and kessler scales. Eur Psychiatry. 2011;26(S2):1689–1689. 10.1016/j.jad.2009.06.020 19615753

[29] WHOQOL-BREF | InfoNTD [Internet]. [cited 2021 Jul 10]. Available from: https://www.infontd.org/toolkits/nmd-toolkit/whoqol-bref

[30] Reba K, Birhane BW, Gutema H. Validity and reliability of the Amharic version of the World Health Organization’s quality of life questionnaire (WHOQOL-BREF) in patients with diagnosed type 2 diabetes in Felege Hiwot referral hospital, Ethiopia. J Diabetes Res. 2019;2019. doi: 10.1155/2019/3513159 31198788 PMC6526560

[31] Eremenco SL, Cella D, Arnold BJ. A comprehensive method for the translation and cross-cultural validation of health status questionnaires. Eval Health Prof. 2005;28(2):212–32. doi: 10.1177/0163278705275342 15851774

[32] Terwee CB, Bot SD, de Boer MR, van der Windt DA, Knol DL, Dekker J, et al. Quality criteria were proposed for measurement properties of health status questionnaires. J Clin Epidemiol. 2007;60(1):34–42. doi: 10.1016/j.jclinepi.2006.03.012 17161752

[33] Salih Mohammed Hassen. Tool validation and psychometric test_Amharic Version of CIASS [Internet]. Mendeley; 2021 [cited 2021 Oct 14]. Available from: https://data.mendeley.com/datasets/gvrk8njd76/1

[34] McHorney CA, Tarlov AR. Individual-patient monitoring in clinical practice: are available health status surveys adequate? Qual Life Res Int J Qual Life Asp Treat Care Rehabil. 1995 Aug;4(4):293–307. doi: 10.1007/BF01593882 7550178

[35] Spadaro G, Romano A, Mosso CO. Contributo alla validazione italiana del ciass. G Ital Med Lav Ergon. 2017 Jan 1;39(1):49–55. 29916621

[36] Browne MW, Cudeck R. Single Sample Cross-Validation Indices for Covariance Structures. Multivar Behav Res. 1989 Oct 1;24(4):445–55. doi: 10.1207/s15327906mbr2404_4 26753509

[37] Hu L, Bentler PM. Cutoff criteria for fit indexes in covariance structure analysis: Conventional criteria versus new alternatives. Struct Equ Model Multidiscip J. 1999 Jan 1;6(1):1–55.

[38] “Structural Equation Modelling: Guidelines for Determining Model Fit” by Daire Hooper, Joseph Coughlan et al. [Internet]. [cited 2021 Mar 29]. Available from: https://arrow.tudublin.ie/buschmanart/2/

[39] Bentler PM. Comparative fit indexes in structural models. Psychol Bull. 1990;107(2):238. doi: 10.1037/0033-2909.107.2.238 2320703

