# Supplementary material for: Translation and psychometric evaluation of chronic illness anticipated stigma scale (CIASS) among patients in Ethiopia
Source: PLoS One. 2022 Jan 21;17(1):e0262744. doi: 10.1371/journal.pone.0262744 (PMC8782391; doi:10.1371/journal.pone.0262744)
Supplement: S1 File — (PDF) [file pone.0262744.s001.pdf]

Questionnaire for Chronic illness anticipated stigma tool cultural translation and validation English questionnaire

CONSENT

Questions for tool validation Information sheet:

Read the statements to the respondent

Purpose of the research project

Dear Participants,

The University of Gondar School of Nursing and principal investigator are conducting the study in three areas of Amhara region referral Hospitals, Ethiopia. Namely University of Gondar Comprehensive Specialized Referral Hospital, Felege-Hiwot Referral Hospitals, and Dessie Referral Hospital. Before conducting the main study we are planned to Translate and culturally validate the research tool in this institution. Also, the overall aim of the study is to evaluate the level and experience of anticipated stigma and associated factors among patients with chronic illness attending follow-up clinics in Amhara Region Referral Hospitals, Ethiopia. You have been chosen randomly to participate in this study. The main aim of this particular part is to translate, culturally adapt, and investigate the psychometric properties to establish an Ethiopian Amharic version of the anticipated stigma scale to be used in patients with chronic illness.

Procedure: To collect our data, we invite you to take part in our project. If you are willing, you need to understand and sign the consent form. We will ask questions related to your chronic illness anticipated stigma.

Risk and/or discomfort/t: By participating in this research project you may feel some discomfort especially on sacrificing your time, otherwise no risk in participating in this study.

Benefits: If you are participating in this research project, the output of the study will have both direct and indirect benefits to you, as well as your family and the community at large will get better treatment and services in the future.

Incentives/payments for participating: You will not be provided any incentives or payment to take part in this project.

Confidentiality: The information collected from this research project will be kept confidential and information about you that will be collected by this study will be stored in a file, without your name, but a code number assigned to it. And it will not be revealed to anyone except the principal investigator and will be kept locked with a key.

Right to refuse or withdraw: You have the full right to refuse from participating in this research. Your refusal will not affect you from getting any kind of health-related service.

Person to contact: If you want to know more information you can contact;

Mr. Mohammed Hassen by Tel: mobile 251-918785735

IRB of UOG by Tel: 251-581141231

If you agree to participate in the study described above, please sign below/ put your finger signature and write the date. Please respond to the interviewer the question in the order. Instructions for how to respond to the different questions will be provided by the interviewer.

I, the undersigned, have understood the objective of the project is "to evaluate the magnitude of anticipated stigma and associated factors among patients with chronic illness attending follow-up clinics in Amhara Region Referral Hospitals, Ethiopia. ", and agreed to be included in the study as explained by the researchers. For this, I also agree to fill the questionnaire which will be interviewed by the interviewer.

Yes ________________ Signature (fingerprint)________________________

No______________Signature (fingerprint)_______________(Terminate the interview)

Date_________________Time started ____________and completed______________

Code Number_________ Area code_____________ Identifier:____________________

Overall information of the participant

**Location and date**		**Response**
1	Interviewer ID	
2	Patient ID	
3	Exact origin/ location for participant	Town_______________ Kebele______________<br>Cell phone (if any) _____________
4	Date of completion of the instrument	DD/MM/YR
5	Time of interview	Hrs_____ Min_____

Instruction:- Please respond to the following question based on the choices you have

Part I. Sociodemographic and institutional characteristics of the study participant

Code	Question	Response	Remarks
101	What is your sex?	1. Male 2. Female	
102	What is your age	_____________Yrs	
103	Residency	1. Urban 2. Rural	
104	Ethnicity	1. Amhara<br>2. Oromo<br>3. Afar<br>4. Tigre<br>5. Other (Pls specify_______)	
105	Religion	1. Orthodox<br>2. Muslim<br>3. Catholic<br>4. Protestant<br>5. Other (Pls specify ______)	
106	Educational Status	1. Not read and write<br>2. Read and write<br>3. primary (1-8 grade)<br>4. Secondary (9-10 grade)<br>5. preparatory (11-12 grade)<br>6. technic schools<br>7. tertiary (diploma and above)	
107	Marital Status	1. Single 2. married<br>3. Separated 4. divorced<br>5. widowed	
108	Occupation	_______________________	
109	Monthly Income	____________________ETB	

110	Access to media related to health	1. No<br>2. Yes	If No pls go to 13
111	Type of media access (More than one response is possible)	1. Tv 3. News paper/magazine<br>2. Radio	
112	Fear of contagion in the hospital	0. No 1. Yes	
113	Attending regular health service	0. No 1. Yes	

Part II:- Clinical and personal Factors

**Code**	**Question**	**Responses**	**Remark**
201	Primary chronic disease diagnosis		
202	Comorbidity	1, No<br>2, Yes	If your response is 0 pls got to Q204
203	How many comorbidities?	______________________	
204	Duration of chronic illness	_____________yrs	
205	Type of medication<br>(You can tick more than one response)	1, Analgesics 2, Cardio-vascular<br>3, Respiratory 4, Endocrine<br>5, Chemotherapy 6, ART<br>7, Other (Pls list____________)	
206	Are you a smoker	1, Non- smokers<br>0. former smokers (ex-smokers)<br>1. Occasional Smoker<br>2. Daily smokers	
207	If daily smoker (4) how many cigarette / day?	______________ Cigarette	
208	Have you ever tried chat chewing?	1. No<br>2. Yes	If No pls got to part III
209	Are you currently taking chat	1. No 2. Yes	

Part III. The Chronic Illness Anticipated Stigma Scale (CIASS)

Instructions: The statements listed below describe some of the ways that people who have chronic illnesses are treated by others. Please read these statements and mark how likely you think that they could happen to you in the future.

First, think about how your friends and family members such as parents, sisters and brothers, and children will treat you in the future. How likely is it that they will treat you in the following way. **Because of your chronic illness?**

S.no	Questions	Very Unlikely	Unlikely	Somewhat likely	Likely	Very Likely	Remark
301	A friend or family member will be angry with you.	1	2	3	4	5	
302	A friend or family member will blame you for not getting better.	1	2	3	4	5	
303	A friend or family member will think that your illness is your fault.	1	2	3	4	5	
304	A friend or family member will not think as highly of you.	1	2	3	4	5	

Now, think about how your coworkers and employers will treat you in the future. If you are not currently employed, think about coworkers and employers that you might have in the future. How likely is it that they will treat you in the following ways **because of your chronic illness?**

S.no	Question	Very Unlikely	Unlikely	Somewhat likely	Likely	Very Likely	Remark
305	Your employer will not promote you.	1	2	3	4	5	
306	Someone at work will discriminate against you.	1	2	3	4	5	
307	Your employer will assign a challenging project to someone else.	1	2	3	4	5	

308	Someone at work will think that you cannot fulfill your work responsibilities	1	2	3	4	5	

Finally, think about how healthcare providers such as doctors, nurses, technicians, and secretaries who work at hospitals and doctor's offices will treat you in the future. How likely is it that they will treat you in the following ways **because of your chronic illness?**

S.no		Very Unlikely	Unlikely	Somewhat likely	Likely	Very Likely	Remark
309	A health care worker will be frustrated with you.	1	2	3	4	5	
310	A health care worker will give you poor care.	1	2	3	4	5	
311	A health care worker will blame you for not getting better.	1	2	3	4	5	
312	A health care worker will think that you are a bad patient.	1	2	3	4	5	

Part IV. Kessler psychological distress scale (CMD)

These questions concern how you have been feeling over the **past 30 days**. Tick a box below each question that best represents how you have been.

S.no	Question	None of the time	A little of the time	Some of the Time	Most of the time	All of the time	Remark
401	During the last 30 days, about how often did you feel tired out for no good reason?	1	2	3	4	5	
402	During the last 30 days, about how often did you feel nervous?	1	2	3	4	5	
403	During the last 30 days, about how often did you feel so nervous that	1	2	3	4	5	

	nothing could calm you down?						
404	During the last 30 days, about how often did you feel hopeless?	1	2	3	4	5	
405	During the last 30 days, about how often did you feel restless or fidgety?	1	2	3	4	5	
406	During the last 30 days, about how often did you feel so restless you could not sit still?	1	2	3	4	5	
407	During the last 30 days, about how often did you feel depressed?	1	2	3	4	5	
408	During the last 30 days, about how often did you feel that everything was an effort?	1	2	3	4	5	
409	During the last 30 days, about how often did you feel so sad that nothing could cheer you up?	1	2	3	4	5	
410	During the last 30 days, about how often did you feel worthless?	1	2	3	4	5	

Part V. WHOQOL- BREF

The following questions ask how you feel about your quality of life, health, or other areas of your life. I will read out each question to you, along with the response options. **Please choose the answer that appears most appropriate.** If you are unsure about which response to give to a question, the first response you think of is often the best one.

Please keep in mind your standards, hopes, pleasures, and concerns. We ask that you think about your life **in the last four weeks.**

S.No	Question	Very poor	Poor	Neither poor nor good	Good	Very good	Remark
501	How would you rate your quality of life?	1	2	3	4	5	

S.No	Question	Very dissatisfied	Dissa tisfied	Neither satisfied nor dissatisfied	Satisfied	Very satisfied	Rem ark
502	How satisfied are you with your health?	1	2	3	4	5	

The following questions ask about **how much** you have experienced certain things in the **last four weeks.**

S.no	Question	Not at all	A little	A moderate amount	Very much	An extreme amount	Remar k
503	To what extent do you feel that physical pain prevents you from doing what you need to do?	1	2	3	4	5	
504	How much do you need any medical treatment to function in your daily life?	1	2	3	4	5	
505	How much do you enjoy life?	1	2	3	4	5	
506	To what extent do you feel your life to be meaningful?	1	2	3	4	5	
507	How well are you able to concentrate?	1	2	3	4	5	

S.no	Question	Not at all	A little	A moderate amount	Very much	Extremely	Remar k
508	How safe do you feel in your daily life?	1	2	3	4	5	
509	How healthy is your physical environment?	1	2	3	4	5	

The following questions ask about how completely you experience or were able to do certain things in **the last four weeks.**

S.no	Question	Not at all	A little	Moderately	Mostly	Completely	Remark

510	Do you have enough energy for everyday life?	1	2	3	4	5	
511	Are you able to accept your bodily appearance?	1	2	3	4	5	
512	Have you enough money to meet your needs?	1	2	3	4	5	
513	How available to you is the information that you need in your day-to-day life?	1	2	3	4	5	
514	To what extent do you have the opportunity for leisure activities?	1	2	3	4	5	

**S.NO**	**Question**	**Very poor**	**Poor**	**Neither poor nor good**	**Good**	**Very good**	**Remark**
515	How well are you able to get around?	1	2	3	4	5	

**S.no**	**Questions**	**Very dissatisfied**	**Dissatisfied**	**Neither satisfied nor dissatisfied**	**Satisfied**	**Very satisfied**	**Remark**
516	How satisfied are you with your sleep?	1	2	3	4	5	
517	How satisfied are you with your ability to perform your daily living activities?	1	2	3	4	5	
518	How satisfied are you with your capacity for work?	1	2	3	4	5	
519	How satisfied are you with yourself?	1	2	3	4	5	
520	How satisfied are you with your personal relationships?	1	2	3	4	5	
521	How satisfied are you with your sex life?	1	2	3	4	5	

522	How satisfied are you with the support you get from your friends?	1	2	3	4	5	
523	How satisfied are you with the conditions of your living place?	1	2	3	4	5	
524	How satisfied are you with your access to health services?	1	2	3	4	5	
525	How satisfied are you with your transport?	1	2	3	4	5	

The following question refers to how often you have felt or experienced certain things in

the last four weeks

**S. No**	**Question**	**Never**	**Seldom**	**Quite often**	**Very often**	**Always**	**Remark**
526	How often do you have negative feelings such as blue mood, despair, anxiety, depression?	1	2	3	4	5	

Thank you for your time and responses
