# Supplementary material for: Translation and psychometric evaluation of chronic illness anticipated stigma scale (CIASS) among patients in Ethiopia
Source: PLoS One. 2022 Jan 21;17(1):e0262744. doi: 10.1371/journal.pone.0262744 (PMC8782391; doi:10.1371/journal.pone.0262744)
Supplement: Attachment — Submitted filename: Comments.docx [file pone.0262744.s003.docx]

Thanks for the opportunity to review this manuscript. The authors exploratorily translated the Chronic Illness Anticipated Stigma Scale (CIASS) into the Amharic language and assessed the reliability and validity of the scale in patients with chronic illness. Although, the findings do provide useful tool for scale localization. I have many concerns with the paper in its current form. I describe these concerns below, separating them into different parts.

Introduction:

1. Please adjust the structure of the Introduction. Why there are only two sentences in the first paragraph?

2. Some conclusion seems needing citations, such as: the second sentence in the 2^nd^ paragraph.

3. The introduction is too long to introduce the backgrounds for the authors. It is not needed to have a literature review for an article, and the authors just introduced the research progress in this field.

4. Please add the aims of the current study in the last paragraph in the Introduction

Methods:

1. Some basic information was not shown in the Participants part. Please introduce the details of the inclusive and exclusive criteria. How do the authors define the chronic patients, and what chronic disorders were included?

2. Why do the authors introduce the related factors of stigma in the Methods? Such as the 2^nd^ paragraph in Variable and Measures. Please adjust the content.

3. Why do the authors select 13 patients to do cognitive interviews?

4. Analytical process: the authors should introduce the details of the primary statistical analysis process. Moreover, have the authors run other models for factor analysis, and why do the authors choose PCA?

5. Please add the inter-rater correlation coefficient of the scale score after two-day training for the collectors.

Results:

1. Please add abbreviations for each Table and Fig.

2. Please adjust the background of the two Figs, maybe the white background is better.

3. Some contents in this part are not suitable, which are better in the Methods.

Discussion:

Discussion is too short. Many points need to explore in depth.
