# Supplementary material for: Translation and psychometric evaluation of chronic illness anticipated stigma scale (CIASS) among patients in Ethiopia
Source: PLoS One. 2022 Jan 21;17(1):e0262744. doi: 10.1371/journal.pone.0262744 (PMC8782391; doi:10.1371/journal.pone.0262744)
Supplement: Attachment — Submitted filename: responses for reviewer.docx [file pone.0262744.s004.docx]

Dear Professor :

We are very grateful to you for your precious time and constructive comments on our manuscript. we will do our best for the scientific community to add a body of knowledge to our expertise.

we have tried to revise all the documents based on your important and valuable scientific comments.

For each point of your comments and suggestions, the responses are available below.

Table 2. Comments and responses for the external reviewer

| S.no | Section | Comments /concerns raised | Responses | Remarks |
| --- | --- | --- | --- | --- |
| 01 | Introduction | Please adjust the structure of the Introduction. Why there are only two sentences in the first paragraph? | Thank you. we have adjusted. | Accepted the changes |
|  |  | Some conclusion seems to need citations, such as the second sentence in the 2^nd^ paragraph | Thank you and we revised based on your suggestion. | In our view better in all together.  We add references to each sentence. |
|  |  | The introduction is too long to introduce the backgrounds of the authors. It is not needed to have a literature review for an article, and the authors just introduced the research progress in this field. | Thank you. we agree with this comment and have revised the section based on your comment. | Accepted the concerns |
|  |  | Please add the aims of the current study in the last paragraph in the Introduction | Thank you. we agree with this suggestion and have incorporated your suggestion in the last introduction section. | Accepted the concerns |
| 02 | Methods | Some basic information was not shown on the Participant's part. Please introduce the details of the inclusive and exclusive criteria. How do the authors define the chronic patients, and what chronic disorders were included? | Thank you for pointing this out. We have added more clarification at the inclusion and exclusion criteria. The definition of chronic illness and disease have also included the type of disease incorporated in our study | Thank you we accept the concerns. |
|  |  | Why do the authors introduce the related factors of stigma in the Methods? Such as the 2^nd^ paragraph in Variable and Measures. Please adjust the content. | Thank you. You have raised an important point here. However, the main reason we have incorporated the associated factors was to show how much our tool would line up with other concepts.  Among different types of validity, a convergent validity test was done with those variables and we choose them. Because most scholars advised using different measurements to see how much our tool addressed the concept and valid. | Thank you for your concern.  No change was done except to merge the second and third paragraphs. |
|  |  | Why do the authors select 13 patients to do cognitive interviews? | Thank you for your critical insights about the pretest. Different scholars recommend pretesting (Cognitive interview) from 5% to 10% of the total proposed sample. So, we have chosen in the middle which is 7.5%. However, if it lacks scientific sound we can put it on the limitation section. | Thank you for the comment, no change. |
|  |  | Analytical process: the authors should introduce the details of the primary statistical analysis process. Moreover, have the authors run other models for factor analysis, and why do the authors choose PCA? | Thank you. You have raised an important point here.  We have used the most advisable method of statistical analysis for the CIASS based on the main tool developer technique.  Moreover, the main reason we chose CFA over EFA was that EFA is for Exploring the new tool development, but our tool has developed and we did only translation to the Amharic language. | hank you. You have raised an important point here. |
|  |  | Please add the inter-rater correlation coefficient of the scale score after two-day training for the collectors. | You have raised an important point. Do you mean the Intraclass correlation coefficient among data collectors? If so, for the objective of inter-rater reliability, we did an intraclass correlation coefficient among 36 patients and it shows 0.875 average measures with 95% confidence intervals (0.755, 0.936). | We add Intraclass correlation coefficient at the method section |
| 03 | Results | Please add abbreviations for each Table and Fig. | Thank you. we have corrected it. | Accept the comment |
|  |  | Please adjust the background of the two Figs, maybe the white background is better | Thank you. we have changed it to a white background for structural equation model diagrams | Accept the comment |
|  |  | Some contents in this part are not suitable, which are better in the Methods | Dear professor, we are not clear which content needs to be put in the method section. However, we tried our best to fitted each section based on the guideline of the journal. | Thank you |
| 04 | Discussion | The discussion is too short. Many points need to explore in-**depth** | Thank you for this suggestion. It would have been interesting to explore this aspect. However, the tool has not been translated to many areas and cultures and we didn’t get any more for discussion. Therefore, to make it more precise and clear we have tried to summarize findings**.** | Thank you |
| 05 | Grammatical issue | Need grammar revision | Thank you professor, we revised all contents by consulting an English language professionals. |  |

Thank you so much for your valuable and important comments and suggestions.
