# Supplementary material for: Translation and psychometric evaluation of chronic illness anticipated stigma scale (CIASS) among patients in Ethiopia
Source: PLoS One. 2022 Jan 21;17(1):e0262744. doi: 10.1371/journal.pone.0262744 (PMC8782391; doi:10.1371/journal.pone.0262744)
Supplement: Attachment — Submitted filename: Dear Editors.docx [file pone.0262744.s005.docx]

**Dear Editors**

Thank you for an excellent opportunity to involve in such an important scientific journal to be one of the selected manuscripts to this stage.

Here, I will try to elaborate on each raised comment and issues

1. In the Discussion section, the authors should summarize their observations and how they were similar or different from prior studies. The editor would expect to see some points regarding how to translate these observations to help address this public health concern.

**Response** – Thank you for your excellent insight and important consideration in the discussion section. We tried to incorporate the new points which will add to compare and contrast our findings. Also, in the conclusion and discussion section, we tried to add points on the scientific merit.

2. If applicable, we recommend that you deposit your laboratory protocols in [protocols.io](http://protocols.io) to enhance the reproducibility of your results.

**Response** – It was an excellent reminding, but our study doesn’t have any protocols. Except for our main data which was attached in the Mendeley Data Depository (DOI 0.17632/gvrk8njd76.1).

**Journal Requirements**

1. Please review your reference list to ensure that it is complete and correct. If you have cited papers that have been retracted, please include the rationale for doing so in the manuscript text, or remove these references and replace them with relevant current references. Any changes to the reference list should be mentioned in the rebuttal letter that accompanies your revised manuscript. If you need to cite a retracted article, indicate the article’s retracted status in the References list and also include a citation and full reference for the retraction notice.

**Response** – We thank you for the comments related to the reference section. Dear editor before we submit to the journal used Zotero reference software and we ask for an excuse to our reference mix arrangement. We attached in the next Table 1 for each change and reason to change.

Table 1. List of references that were revised based on the current manuscript.

| **S.no** | **Previous reference list** | **New reference list** | **Reasons for change** |
| --- | --- | --- | --- |
| 01 | Ref 1 & 2 | No change | |
| 02 | Ref 3 | Change done | Revised based on the current source from free internet |
| 03 | Ref 4 up to 10 | No change | |
| 04 | Ref 11 | Removed | It was redundant with reference number 04 |
| 05 | Ref 12 up to 15 | Changed to 11 up to 14 | Reference order based |
| 06 | Ref 16 | Reference 15 | Edited the author list |
| 07 | Ref 17 | Reference 16 | All parts edited based on the current internet availability |
| 08 | Ref 18 &19 | Changed to 17 & 18 | Based on reference order |
| 09 | Ref 20 (Removed) | Added new ref 19 (Trejos-Herrera A. et.al) | We found the recent literature which supported our paper. |
| 10 | Ref 20 up to Ref 25 | No change | |
| 11 | Added new ref 26 | | This reference supported our K10 psychological distress scale |
| 12 | Ref 26 up to ref 33 | Changed to ref 27 up to ref 34 | Based on reference order |
| 13 | Added new Ref 35 | | It was one of the supportive papers we missed in the previous literature. |
| 14 | Ref 34 up to 37 | Changed to ref 36 up to Ref 39 | Based on the reference order |

2. While revising your submission, please upload your figure files to the Preflight Analysis and Conversion Engine (PACE) digital diagnostic tool.

**Response –** thank you for your notification on the standard digital diagnostic tool. Created the account and got better picture quality. Uploaded our picture (Fig 1 & Fig 2).

**We thank you for our repeated conversation and support**
